# Management of unspecified anxiety disorder: Expert consensus

**DOI:** 10.1002/npr2.12323

**Published:** 2023-02-21

**Authors:** Hitoshi Sakurai, Ken Inada, Yumi Aoki, Masahiro Takeshima, Kenya Ie, Morito Kise, Eriko Yoshida, Takashi Tsuboi, Hisashi Yamada, Hikaru Hori, Yasushi Inada, Eiji Shimizu, Kazuo Mishima, Koichiro Watanabe, Yoshikazu Takaesu

**Affiliations:** ^1^ Department of Neuropsychiatry Kyorin University Faculty of Medicine Tokyo Japan; ^2^ Department of Psychiatry, School of Medicine Kitasato University Kanagawa Japan; ^3^ Psychiatric and Mental Health Nursing St. Luke's International University Tokyo Japan; ^4^ Department of Neuropsychiatry Akita University Graduate School of Medicine Akita Japan; ^5^ Division of General Internal Medicine, Department of Internal Medicine St. Marianna University School of Medicine Kanagawa Japan; ^6^ Division of General Internal Medicine, Department of Internal Medicine Kawasaki Municipal Tama Hospital Kanagawa Japan; ^7^ Centre for Family Medicine Development Japanese Health and Welfare Co‐operative Federation Tokyo Japan; ^8^ Department of General Internal Medicine, Kawasaki Kyodo Hospital Kawasaki Health Cooperative Association Kanagawa Japan; ^9^ Department of Neuropsychiatry Hyogo Medicial University Hyogo Japan; ^10^ Department of Psychiatry, Faculty of Medicine Fukuoka University Fukuoka Japan; ^11^ Medical corporation YUJIN‐KAI Inada Clinic Osaka Japan; ^12^ Research Center for Child Mental Development Chiba University Chiba Japan; ^13^ Department of Cognitive Behavioral Physiology, Graduate School of Medicine Chiba University Chiba Japan; ^14^ Department of Neuropsychiatry, Graduate School of Medicine University of the Ryukyus Okinawa Japan

**Keywords:** anxiolytics, benzodiazepine, expert consensus, unspecified anxiety disorder

## Abstract

**Aims:**

Treatment guidelines with respect to unspecified anxiety disorder have not been published. The aim of this study was to develop a consensus among field experts on the management of unspecified anxiety disorder.

**Methods:**

Experts were asked to evaluate treatment choices based on eight clinical questions concerning unspecified anxiety disorder using a nine‐point Likert scale (1 = “disagree” to 9 = “agree”). According to the responses from 119 experts, the choices were categorized into first‐, second‐, and third‐line recommendations.

**Results:**

Benzodiazepine anxiolytic use was not categorized as a first‐line recommendation for the primary treatment of unspecified anxiety disorder, whereas multiple nonpharmacological treatment strategies, including coping strategies (7.9 ± 1.4), psychoeducation for anxiety (7.9 ± 1.4), lifestyle changes (7.8 ± 1.5), and relaxation techniques (7.4 ± 1.8), were categorized as first‐line recommendations. Various treatment strategies were categorized as first‐line recommendations when a benzodiazepine anxiolytic drug did not improve anxiety symptoms, that is, differential diagnosis (8.2 ± 1.4), psychoeducation for anxiety (8.0 ± 1.5), coping strategies (7.8 ± 1.5), lifestyle changes (7.8 ± 1.5), relaxation techniques (7.2 ± 1.9), and switching to selective serotonin reuptake inhibitors (SSRIs) (7.0 ± 1.8). These strategies were also highly endorsed when tapering the dosage of or discontinuing benzodiazepine anxiolytic drugs. There was no first‐line recommendation regarding excusable reasons for continuing benzodiazepine anxiolytics.

**Conclusions:**

The field experts recommend that benzodiazepine anxiolytics should not be used as a first‐line option for patients with unspecified anxiety disorder. Instead, several nonpharmacological interventions and switching to SSRIs were endorsed for the primary treatment of unspecified anxiety disorder and as alternatives to benzodiazepine anxiolytics.

## Abstract 2

We developed an expert consensus on the management of unspecified anxiety disorder. Nonpharmacological interventions, including coping strategies, psychoeducation for anxiety, lifestyle changes, and relaxation techniques, were categorized as first‐line recommendations for several situations in unspecified anxiety disorders, whereas benzodiazepine anxiolytic drugs were not recommended for any situations.

## Graphical abstract

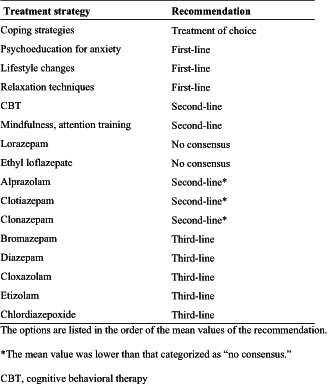

## 1 INTRODUCTION

Anxiety disorders are frequent and persistent illnesses that is regarded as the ninth most health‐related cause of disability.^1^ Treatment guidelines based on solid evidence that mainly stems from randomized controlled trials are available in some anxiety disorders including generalized anxiety disorder, panic disorder, and social anxiety disorder.^2^, ^3^, ^4^, ^5^ Unspecified anxiety disorder is a diagnosis that is characterized as significant anxiety or phobias without the exact criteria for any other anxiety disorders. According to a cross‐sectional study using nationally representative data for physician office‐based visits in the United States, unspecified anxiety disorder was the major diagnosis in anxiety disorders, increasing 50% between 1999 and 2002 to 62% between 2007 and 2010.^6^ However, to our knowledge, there have been no reports of randomized controlled trials for unspecified anxiety disorder probably because of the heterogeneity of this disease. Thus, treatment guidelines for unspecified anxiety disorder have not been published with the result that clinicians always have difficulty in making treatment decision.

Opinions from field experts reflecting actual clinical experience are useful in providing clear treatment recommendations for issues that are clinically relevant but failed to be addressed in previous studies. Notably, benzodiazepine anxiolytics have been used for the treatment of anxiety disorders due to their efficacy, fast action, and no serious side effects in the short term.^7^, ^8^ While maintenance treatment with benzodiazepines has been considered to be associated with several adverse events, including sedation, cognitive impairments, and potential for dependence,^9^ recent reports indicated that their efficacy and safety could also be maintained in the long term.^8^, ^10^, ^11^, ^12^, ^13^

The aim of the present study was therefore to develop a consensus among experts on the management of unspecified anxiety disorders, including the use of benzodiazepine anxiolytics.

## 2 METHODS

### 2.1 Study design

Eight clinical questions on the management of unspecified anxiety disorder were identified by the task force which consists of 13 mental health professionals and primary care providers. For each question, the available treatment choices were arranged according to the treatment guidelines for other anxiety disorders^2^, ^3^, ^4^, ^5^ and clinical practice. Certified psychiatrists from the Japanese Society of Clinical Neuropsychopharmacology and the Japanese Society of Sleep Research and councilors from the Japanese Society of Anxiety and Related Disorders throughout the country were invited to participate in the questionnaire survey by email from June 29, 2022, to July 31, 2022. The experts who agreed to participate were asked to evaluate each treatment choice using a nine‐point Likert scale (1 = “strongly not recommended” to 9 = “strongly recommended”). The definition of unspecified anxiety disorder according to the Diagnostic and Statistical Manual of Mental Disorders, Fifth Edition, in their native language (i.e., Japanese) was included in the questionnaire. Clinical questions and treatment choices are presented in Table S1. The survey took approximately 15 min to complete. The experts voluntarily participated in the survey, without any incentives. Respondents were also asked to indicate their age range, gender, and affiliated societies. This study was approved by the institutional review board of St. Luke's International University (2021‐604).

### 2.2 Analysis

The following values were calculated for each treatment option: mean, standard deviation, 95% confidence interval (CI), and number of rating categories (i.e., not recommended: responses 1–3; neutral: responses 4–6, and recommended: responses 7–9). Pearson's chi‐squared test was used to compare the numbers of these three rating categories for each treatment choice. When the responses were evenly distributed across the three categories with a *p*‐value ≥0.05, the outcome was regarded as “no consensus” for the corresponding clinical question, indicating a controversial strategy. Treatment options with 95% CI values ≥6.5 were regarded as “first‐line treatments/strategies,” indicating a consensus among the experts for a given situation. Options rated as 9 by more than 50% of the responders were defined as “treatments of choice,” indicating a particularly strong first‐line recommendation. Options with 95% CI values ≥3.5 were regarded as “second‐line treatments/strategies,” indicating reasonable options for patients who do not respond to or cannot tolerate the first‐line strategies. Treatment options with 95% CI values <3.5 were considered “third‐line treatments/strategies,” indicating inappropriate options in general or those used only when other options were ineffective.

## 3 RESULTS

### 3.1 Participant characteristics

The questionnaire was completed by 119 experts, which was considered a sufficient number of respondents. The mean age of the respondents was 52.0 ± 10.2 years. The proportions of men and women were 85.7% and 13.4%, respectively. A total of 78 respondents (65.5%) were certified psychiatrists from the Japanese Society of Clinical Neuropsychopharmacology, 27 (22.7%) were certified psychiatrists from the Japanese Society of Sleep Research, and 20 (16.8%) were councilors from the Japanese Society of Anxiety and Related Disorders.

### 3.2 Primary treatment strategy for unspecified anxiety disorder

Benzodiazepine anxiolytic use was not categorized as a first‐line recommendation for the primary treatment of unspecified anxiety disorder (Table 1). Lorazepam (4.9 ± 2.4) and ethyl loflazepate (4.8 ± 2.2) were categorized as “no consensus,” whereas other benzodiazepine anxiolytics had lower mean values. By contrast, multiple nonpharmacological strategies, including coping strategies (7.9 ± 1.4), psychoeducation for anxiety (7.9 ± 1.4), lifestyle changes (7.8 ± 1.5), and relaxation techniques (7.4 ± 1.8), were categorized as first‐line choices for unspecified anxiety disorder. Coping strategies were categorized as the “treatment of choice” as it was rated with 9 by 50.4% of the respondents.

**TABLE 1:** Recommended primary treatment strategy for unspecified anxiety disorders.

Treatment strategy	Recommendation
Coping strategies	Treatment of choice
Psychoeducation for anxiety	First‐line
Lifestyle changes	First‐line
Relaxation techniques	First‐line
CBT	Second‐line
Mindfulness, attention training	Second‐line
Lorazepam	No consensus
Ethyl loflazepate	No consensus
Alprazolam	Second‐line^a^
Clotiazepam	Second‐line^a^
Clonazepam	Second‐line^a^
Bromazepam	Third‐line
Diazepam	Third‐line
Cloxazolam	Third‐line
Etizolam	Third‐line
Chlordiazepoxide	Third‐line

*Note*: The options are listed in the order of the mean values of the recommendation.

Abbreviation: CBT, cognitive behavioral therapy.

^a^ The mean value was lower than that categorized as “no consensus.”

### 3.3 Treatment strategy when benzodiazepine anxiolytics are ineffective

When benzodiazepine anxiolytic drug use did not improve anxiety symptoms, the only first‐line pharmacological treatment was switching to selective serotonin reuptake inhibitors (SSRIs) (7.0 ± 1.8) (Table 2). Switching to other antidepressants, including serotonin and norepinephrine reuptake inhibitors (5.7 ± 2.0) and mirtazapine (5.5 ± 2.1), was categorized as second‐line treatments. However, several treatment strategies were highly recommended in the case of no improvement with benzodiazepine anxiolytics. Differential diagnosis (8.2 ± 1.4, rated with 9 by 63.0% of the respondents) and psychoeducation for anxiety (8.0 ± 1.5, rated with 9 by 51.3% of the respondents) were categorized as “treatments of choice,” whereas coping strategies (7.8 ± 1.5), lifestyle changes (7.8 ± 1.5), and relaxation techniques (7.2 ± 1.9) were categorized as first‐line recommendations.

**TABLE 2:** Recommended treatment strategy when treatment with benzodiazepine anxiolytic drugs does not improve the symptoms of unspecified anxiety disorders.

Treatment strategy	Recommendation
Differential diagnosis	Treatment of choice
Psychoeducation for anxiety	Treatment of choice
Coping strategies	First‐line
Lifestyle changes	First‐line
Relaxation techniques	First‐line
Switching to SSRIs	First‐line
CBT	Second‐line
Referral to a specialist hospital	Second‐line
Mindfulness, attention training	Second‐line
Switching to SNRI	Second‐line
Switching to mirtazapine	Second‐line
Switching to tandospirone	Second‐line
Switching to another benzodiazepine anxiolytic drug	No consensus
Switching to an antipsychotic drug	Second‐line^a^
Increasing the dose of an anxiolytic drug	Second‐line^a^
Switching to Kampo	Second‐line^a^
Switching to an antiepileptic drug	Third‐line
Combination of two benzodiazepine anxiolytics	Third‐line
Switching to an antihistaminic drug	Third‐line

*Note*: The treatment strategies are listed in order of the mean values of the recommendation.

Abbreviations: CBT, cognitive behavioral therapy; SNRI, serotonin and norepinephrine reuptake inhibitor; SSRI, selective serotonin reuptake inhibitor.

^a^ The mean value was lower than that categorized as “no consensus.”

### 3.4 Discontinuation of benzodiazepine anxiolytic drugs

There was no first‐line recommendation for the timing of dosage tapering or discontinuation of a benzodiazepine anxiolytic drug after the improvement of anxiety symptoms. Notably, 1–3 month(s) (5.7 ± 2.2) and 3–6 months (5.7 ± 2.1) after the improvement of symptoms were categorized as second‐line recommendations. Moreover, there was also no first‐line recommendation concerning excusable reasons for continuing benzodiazepine anxiolytic drugs. All suggested clinical reasons involving anticipation of physical or mental deterioration (6.7 ± 2.0), history of relapsed anxiety symptoms (6.7 ± 2.1), and no stabilization of physical or mental states or quality of life (5.9 ± 2.0) were categorized as second‐line recommendations. Various strategies were highly recommended when tapering the dosage of or discontinuing benzodiazepine anxiolytic drugs: gradual reduction (8.1 ± 1.2), psychoeducation for anxiety (7.8 ± 1.5), lifestyle changes (7.7 ± 1.4), coping strategies (7.7 ± 1.5), and relaxation techniques (7.1 ± 2.0) (Table 3). Switching to SSRIs (7.0 ± 2.0) was highly recommended when tapering the dosage of or discontinuing benzodiazepine anxiolytic drugs.

**TABLE 3:** Recommended treatment strategy for tapering the dosage of or discontinuing a benzodiazepine anxiolytic drug for unspecified anxiety disorders.

Treatment strategy	Recommendation
Gradual reduction	First‐line
Psychoeducation for anxiety	First‐line
Lifestyle changes	First‐line
Coping strategies	First‐line
Relaxation techniques	First‐line
Switching to SSRIs	First‐line
CBT	Second‐line
Mindfulness, attention training	Second‐line
Switching to PRN	Second‐line
Switching to another drug	Second‐line
Switching to SNRIs	Second‐line
Switching to mirtazapine	Second‐line
Switching to tandospirone	Second‐line
Self‐management	No consensus
Switching to Kampo	No consensus
Switching to an antipsychotic drug	Second‐line^a^
Switching to an antiepileptic drug	Third‐line
Switching to an antihistaminic drug	Third‐line

*Note*: The treatment strategies are listed in order of the mean values of the recommendation.

Abbreviations: CBT, cognitive behavioral therapy; PRN, pro re nata (as needed); SNRI, serotonin and norepinephrine reuptake inhibitor; SSRI, selective serotonin reuptake inhibitor.

^a^ The mean value was lower than that categorized as “no consensus.”

## 4 DISCUSSION

In the present study, the practical management of unspecified anxiety disorder was evaluated by the field experts. Benzodiazepine anxiolytic drug use was not considered a first‐line strategy for the primary treatment in unspecified anxiety disorder and was only recommended for short‐term use after symptom improvement. By contrast, nonpharmacological strategies were highly endorsed for several situations, including primary treatment, subsequent treatment for no improvement with a benzodiazepine anxiolytic drug, and as an alternative to treatment with a benzodiazepine anxiolytic drug. In addition, switching to SSRIs was strongly recommended when tapering the dosage of or discontinuing benzodiazepine anxiolytic drugs. These recommendations reflect the experts' consensus on reducing the use of benzodiazepine anxiolytic drugs as much as possible in patients with unspecified anxiety disorder.

Benzodiazepine anxiolytics use was not recommended as a first‐line strategy for the treatment of unspecified anxiety disorder; however, lorazepam and ethyl loflazepate, which were categorized as “no consensus,” were the most commonly endorsed benzodiazepines. This may reflect physicians' efforts to avoid prescribing benzodiazepine anxiolytic drugs, possibly because of the potential adverse events. In the principle of “above all, do no harm,” benzodiazepine anxiolytics should be limited to the treatment of specified anxiety disorders, including generalized anxiety disorder, panic disorder, and social anxiety disorder, for which benzodiazepine anxiolytics are evidenced to be somewhat effective.^3^, ^8^ By contrast, several practical nonpharmacological strategies, including coping strategies, psychoeducation for anxiety, lifestyle changes, and relaxation techniques, were categorized as first‐line recommendations for the primary treatment of unspecified anxiety disorder. Coping strategies were categorized as the “treatment of choice.” Coping strategies may improve anxiety symptoms due to life events, alleviate stress, and promote positive psychological outcomes.^14^, ^15^, ^16^, ^17^ Further investigations are needed to clarify the types of coping strategies appropriate for this population.

Similar to the primary treatment of unspecified anxiety disorder, several practical nonpharmacological strategies, including differential diagnosis, psychoeducation for anxiety, coping strategies, lifestyle changes, and relaxation techniques, were categorized as first‐line recommendations in cases of nonimprovement with a benzodiazepine anxiolytic drug. It is noteworthy that these nonpharmacological strategies were endorsed more than any other pharmacological strategy. Among them, differential diagnosis and psychoeducation for anxiety were categorized as “treatments of choice.” Physicians should thoroughly assess patients' time course and diagnose diseases, including anxiety disorders due to other illnesses or causes (e.g., excessive caffeine intake).^8^ They should also consider passive (i.e., single leaflets, emails, or websites) or active (i.e., multisession group interventions with exercises and therapist guidance) education of patients to increase their knowledge, better cope with symptoms, and reduce psychological distress.^18^ Regarding pharmacological strategies for nonimprovement with a benzodiazepine anxiolytic drug, only switching to SSRIs was categorized as a first‐line treatment, possibly because some SSRIs have been approved for anxiety disorders. While there is no evidence for the superiority of newer antidepressants over benzodiazepines in terms of efficacy for anxiety disorders,^8^, ^19^ potential cognitive impairments and dependence on benzodiazepines could be prevented by switching to newer antidepressants. If these nonpharmacological or pharmacological treatment strategies are not effective, physicians should consider referral to a psychiatry hospital/clinic or organized nonpharmacological treatments involving cognitive behavioral therapy, mindfulness, and attention training, which were categorized as second‐line recommendations.

There was no first‐line recommendation for the timing of dosage tapering or discontinuing benzodiazepine anxiolytic drugs after the improvement of anxiety symptoms. A treatment period of 1–6 months may be reasonable for the prevention of symptom relapse and potential long‐term side effects.^9^ There was also no first‐line option for the continuation of benzodiazepine anxiolytic drugs, indicating that benzodiazepine anxiolytics should be discontinued at some point during acute treatment for unspecified anxiety disorder.

Multiple strategies, including gradual reduction, psychoeducation for anxiety, lifestyle changes, coping strategies, and relaxation techniques, were categorized as first‐line recommendations when tapering the dosage of or discontinuing benzodiazepine anxiolytic drugs. While these nonpharmacological treatment strategies are considered useful in preventing relapse, sociodemographic and clinical characteristics related to the prevention of anxiety are unclear.^20^ Further studies are needed to improve the methodological quality and investigate proper strategies depending on individual factors. Similar to the pharmacological strategy in cases of nonimprovement with a benzodiazepine anxiolytic drug, switching to SSRIs was categorized as a first‐line recommendation when tapering the dosage of or discontinuing a benzodiazepine anxiolytic drug. In contrast to this strong recommendation, only paroxetine has been systematically examined for the facilitation of benzodiazepine discontinuation in chronic benzodiazepine users.^21^ Moreover, a meta‐analysis demonstrated that there was no facilitation effect with paroxetine compared with placebo or no intervention.^21^ This lack of evidence should be taken into consideration when determining treatment strategies.

This study had several limitations. First, this is an expert consensus for the management of unspecified anxiety disorder, which is ranked as having a low level of evidence in evidence‐based medicine. Because many recommendations in the present report have not been fully examined, further investigation is required. Second, a lack of adequate information in some clinical questions may have affected the ability of the respondents to choose the appropriate treatment. The heterogeneity of patients should be considered when the recommendations in this expert consensus are applied in clinical practice. Third, the questionnaire did not include a question about the initial recommendation for pharmacological treatments other than benzodiazepine anxiolytics in unspecified anxiety disorders. Fourth, the generalizability of our findings may be limited, considering that all participating experts were involved in Japanese medical care. Finally, the analytical methods and rating categories (i.e., 1–3 [disagree], 4–6 [neutral], and 7–9 [agree]) used in this study were somewhat arbitrary.

In conclusion, the field experts recommend not using benzodiazepine anxiolytics as a first‐line treatment strategy in unspecified anxiety disorder and only prescribing them in the short term, if needed. Instead, several nonpharmacological interventions, as well as switching to SSRIs, were highly endorsed for primary treatment and as alternatives to benzodiazepine anxiolytics. While these recommendations need to be confirmed in future clinical studies, consensus‐based recommendations may be useful to solve clinical questions for which the evidence to date is limited.

## AUTHOR CONTRIBUTIONS

Hitoshi Sakurai contributed to the acquisition and analysis of data, and drafting the manuscript. Ken Inada, Yumi Aoki, Masahiro Takeshima, Kenya Ie, Morito Kise, Eriko Yoshida, Takashi Tsuboi, Hisashi Yamada, Hikaru Hori, Yasushi Inada, Eiji Shimizu, Kazuo Mishima, and Koichiro Watanabe contributed to the acquisition and analysis of data. Yoshikazu Takaesu contributed to the conception and design of the study, and acquisition and analysis of data.

## FUNDING INFORMATION

This study was supported by research grants from the Ministry of Health, Labor and Welfare of Japan (21GC1016).

## CONFLICT OF INTEREST STATEMENT

Dr. Sakurai has received grants from Takeda Science Foundation, and manuscript and speaker fees from Eisai, Takeda Pharmaceutical, Otsuka Pharmaceutical, Meiji Seika Pharma, Shionogi Pharma, Yoshitomiyakuhin, Sumitomo Pharma, Kyowa Pharmaceutical, and Lundbeck Japan. Dr. Ken Inada has received personal fees/grant support from Eisai, Eli Lilly, Janssen, Meiji Seika Pharma, Mitsubishi Tanabe Pharma, Mochida, MSD, Novartis, Otsuka, Shionogi, Sumitomo Pharma, and Yoshitomiyakuhin in the last 3 years. Dr. Aoki declares no conflicts of interest. Dr. Takeshima has received speaker's honoraria from Takeda Pharmaceutical, Otsuka Pharmaceutical, Daiichi Sankyo Company, Sumitomo Pharma, Meiji Seika Pharma, Viatris Pharmaceuticals Japan, MSD, Eisai, Ltd., and Yoshitomi Pharmaceutical and research grants from Otsuka Pharmaceutical, Eisai, Shionogi, and the Japanese Ministry of Health, Labour and Welfare (R3‐21GC1016) outside the submitted work. Dr. Ie has received speaker's honoraria from Eisai and research grants from the Japanese Ministry of Health, Labour and Welfare (18K15434, 22K15678) and the Japan Agency for Medical Research and Development (21fk0108486h0001) outside the submitted work. Dr. Kise declares no conflicts of interest. Dr. Yoshida declares no conflicts of interest. Dr. Tsuboi has received personal fees from Eisai, Kyowa Pharmaceutical Industry, Meiji Seika Pharma, Mitsubishi Tanabe Pharma, Mochida, MSD, Otsuka, Shionogi, Sumitomo Pharma, Takeda Pharmaceutical, Viatris, and Yoshitomiyakuhin within the last 3 years. Dr. Yamada has received speaker's honoraria from Eisai, Eli Lilly, Kyowa Pharmaceutical Industry, Meiji Seika Pharma, Mitsubishi Tanabe Pharma, Mochida, Otsuka, Sumitomo Pharma, Takeda Pharmaceutical, Viatris, and Yoshitomiyakuhin within the last 3 years. Dr. Hori has received speaker's honoraria from Eisai, Eli Lilly, Janssen Pharmaceutical, Meiji Seika Pharma, Mitsubishi Tanabe Pharma, MSD, Otsuka Pharmaceutical, Shionogi, Sumitomo Pharma, Viatris, and Takeda Pharmaceutical. Dr. Yasushi Inada has received speaker's honoraria from Eisai, Eli Lilly, Meiji Seika Pharma, MSD, Sumitomo Pharma, Takeda Pharmaceutical, Viatris Pharmaceuticals Japan, and Yoshitomi Pharmaceutical. Dr. Shimizu has received speaker's honoraria from Mochida Pharmaceutical, KYOWA Pharmaceutical Industry, Astellas, Kyorin, and Dainippon Pharma, and research funding from Sumitomo Pharma outside the submitted work. Dr. Mishima has received speaker's honoraria from EISAI, Nobelpharma, Takeda Pharmaceutical, and MSD. Dr. Mishima has received research grants from Eisai, Sumitomo Pharma, Takeda Pharmaceutical, AMED (JP21dk0307103KM), and the Japanese Ministry of Health, Labour and Welfare (19GC1012 and 21GC0801). Dr. Watanabe has received manuscript fees or speaker's honoraria from Eisai, Eli Lilly, Janssen Pharmaceutical, Kyowa Pharmaceutical, Lundbeck Japan, Meiji Seika Pharma, Mitsubishi Tanabe Pharma, MSD, Otsuka Pharmaceutical, Pfizer, Shionogi, Sumitomo Pharma, and Takeda Pharmaceutical and received research/grant support from Daiichi Sankyo, Eisai, Meiji Seika Pharma, Mitsubishi Tanabe Pharma, MSD, Otsuka Pharmaceutical, Pfizer, Sumitomo Pharma, and Takeda Pharmaceutical. In addition, Dr. Watanabe is a consultant of Boehringer Ingelheim, Daiichi Sankyo, Eisai, Eli Lilly, Janssen Pharmaceutical, Kyowa Pharmaceutical, Lundbeck Japan, Luye Pharma, Mitsubishi Tanabe Pharma, Otsuka Pharmaceutical, Pfizer, Sumitomo Dainippon Pharma, Taisho Toyama Pharmaceutical, and Takeda Pharmaceutical. Dr. Takaesu has received lecture fees from Takeda Pharmaceutical, Sumitomo Dainippon Pharma, Otsuka Pharmaceutical, Meiji Seika Pharma, Kyowa Pharmaceutical, Eisai, MSD, Yoshitomi, and research funding from Otsuka Pharmaceutical, Meiji Seika Pharma, MSD, and Eisai.

## ETHICS STATEMENT

Approval of the Research Protocol by an Institutional Reviewer Board: This study was approved by the institutional review board of St. Luke's International University (2021‐604).

Patient Consent Statement: n/a.

Permission to Reproduce Material from Other Sources: We own all the rights in the material submitted and agree to transfer, assign, or otherwise convey all copyright ownership to the Neuropsychopharmacology Reports upon acceptance of this manuscript.

Registry and the Registration No. of the study/trial: n/a.

## Supporting information


Table S1.

## Data Availability

The data that support the findings of this study are openly available in Figshare at https://doi.org/10.6084/m9.figshare.21909051.v1.
