# Supplementary material for: Management of unspecified anxiety disorder: Expert consensus
Source: Neuropsychopharmacol Rep. 2023 Feb 21;43(2):188–94. doi: 10.1002/npr2.12323 (PMC10275278; doi:10.1002/npr2.12323)
Supplement: Supplementary file 1 — Table S1. [file NPR2-43-188-s001.docx]

Supplementary Table 1. Questionnaire and responses

In this questionnaire, we inquire about the treatment of unspecified anxiety disorders. Please rate the recommended levels for each option.

1 2 3 4 5 6 7 8 9

←Strongly recommend　 Strongly recommend→

Q1) Which benzodiazepine anxiolytics would you recommend for unspecified anxiety disorders?

|  | 95% CI | | | Mean (SD) |
| --- | --- | --- | --- | --- |
|  | Third-line | Second-line | First-line |  |
| 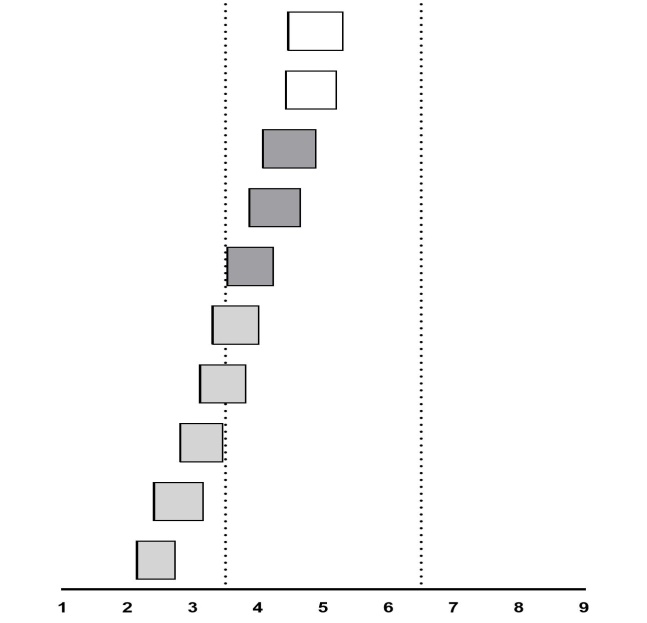Lorazepam |  | | | 4.9 (2.4) |
| Ethyl loflazepate |  | | | 4.8 (2.2) |
| Alprazolam |  | | | 4.5 (2.3) |
| Clotiazepam |  | | | 4.3 (2.2) |
| Clonazepam |  | | | 3.9 (2.0) |
| Bromazepam |  | | | 3.7 (2.0) |
| Diazepam |  | | | 3.5 (2.0) |
| Cloxazolam |  | | | 3.1 (1.9) |
| Etizolam |  | | | 2.8 (2.1) |
| Chlordiazepoxide |  | | | 2.4 (1.7) |

CI, confidence interval; SD, standard deviation

Q2) Which non-pharmacological treatments would you recommend for unspecified anxiety disorders?

|  | 95% CI | | | Mean (SD) |
| --- | --- | --- | --- | --- |
|  | Third-line | Second-line | First-line |  |
| 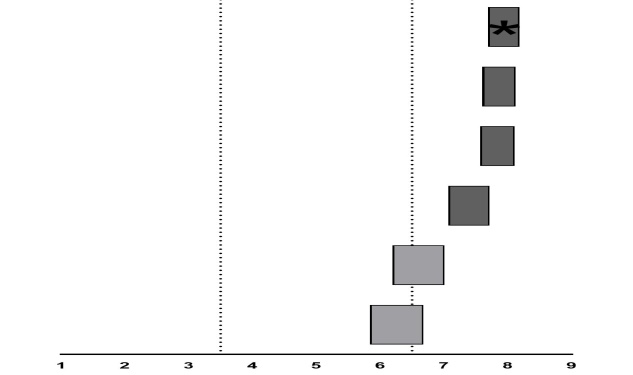Coping strategies |  | | | 7.9 (1.4) |
| Psychoeducation for anxiety |  | | | 7.9 (1.4) |
| Lifestyle changes |  | | | 7.8 (1.5) |
| Relaxation techniques |  | | | 7.4 (1.8) |
| CBT |  | | | 6.6 (2.2) |
| Mindfulness, Attention training |  | | | 6.3 (2.3) |

CBT, cognitive behavioral therapy; CI, confidence interval; SD, standard deviation

Q3) Which pharmacological treatments would you recommend for unspecified anxiety disorders if a benzodiazepine anxiolytic drug did not improve anxiety symptoms?

| 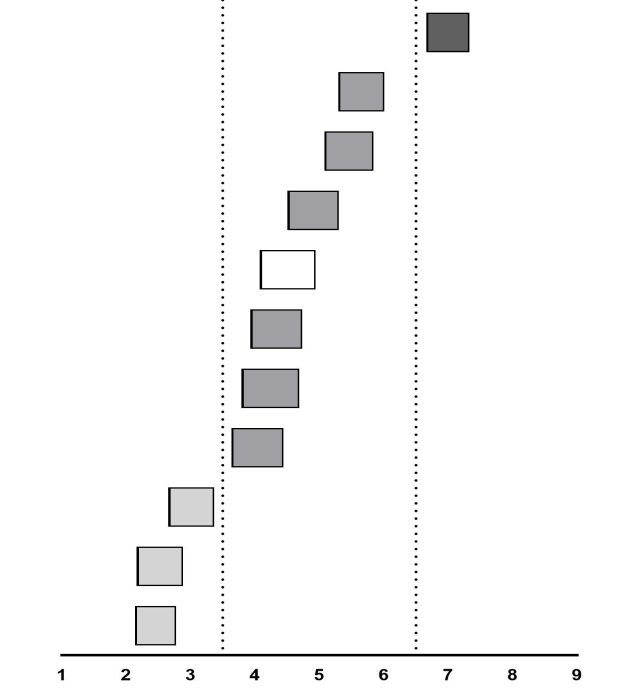 | 95% CI | | | Mean (SD) |
| --- | --- | --- | --- | --- |
|  | Third-line | Second-line | First-line |  |
| Switching to SSRIs |  | | | 7.0 (1.8) |
| Switching to SNRIs |  | | | 5.7 (2.0) |
| Switching to mirtazapine |  | | | 5.5 (2.1) |
| Switching to tandospirone |  | | | 4.9 (2.2) |
| Switching to another benzodiazepine anxiolytic drug |  | | | 4.5 (2.4) |
| Switching to an antipsychotic drug |  | | | 4.3 (2.2) |
| Increasing the dose of an anxiolytic drug |  | | | 4.2 (2.4) |
| Switching to Kampo |  | | | 4.0 (2.2) |
| Switching to an antiepileptic drug |  | | | 3.0 (1.9) |
| Combination of two benzodiazepine anxiolytics |  | | | 2.5 (2.0) |
| Switching to an antihistaminic drug |  | | | 2.5 (1.7) |

CI, confidence interval; SD, standard deviation; SNRI, serotonin and norepinephrine reuptake inhibitor; SSRI, selective serotonin reuptake inhibitor

Q4) Which management or non-pharmacological treatments would you recommend for unspecified anxiety disorders if a benzodiazepine anxiolytic drug did not improve anxiety symptoms?

| 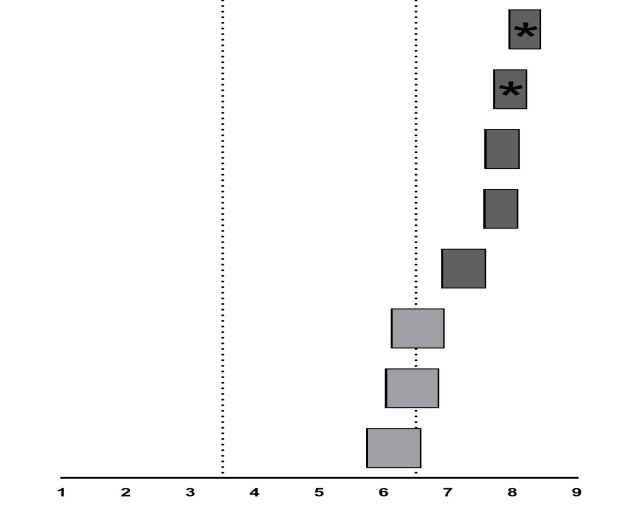 | 95% CI | | | Mean (SD) |
| --- | --- | --- | --- | --- |
|  | Third-line | Second-line | First-line |  |
| Differential diagnosis |  | | | 8.2 (1.4) |
| Psychoeducation for anxiety |  | | | 8.0 (1.5) |
| Coping strategies |  | | | 7.8 (1.5) |
| Lifestyle changes |  | | | 7.8 (1.5) |
| Relaxation techniques |  | | | 7.2 (1.9) |
| CBT |  | | | 6.5 (2.3) |
| Referral to a specialist hospital |  | | | 6.4 (2.3) |
| Mindfulness, attention training |  | | | 6.2 (2.4) |

CBT, cognitive behavioral therapy; CI, confidence interval; SD, standard deviation

Q5) When would you taper the dosage of or discontinue a benzodiazepine anxiolytic drug after the anxiety symptoms improve?

| 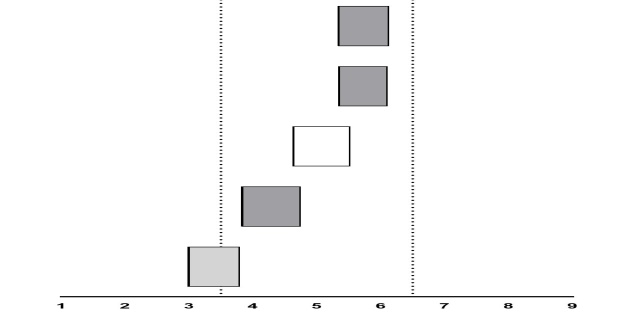 | 95% CI | | | Mean (SD) |
| --- | --- | --- | --- | --- |
|  | Third-line | Second-line | First-line |  |
| After 1–3 month(s) |  | | | 5.7 (2.2) |
| After 3–6 months |  | | | 5.7 (2.1) |
| After 6–12 months |  | | | 5.1 (2.5) |
| Immediately after improvement |  | | | 4.3 (2.6) |
| After more than 12 months |  | | | 3.4 (2.3) |

CI, confidence interval; SD, standard deviation

Q6) Which of the following factors would you consider excusable reasons to continue a benzodiazepine anxiolytic drug?

|  | 95% CI | | | Mean (SD) |
| --- | --- | --- | --- | --- |
|  | Third-line | Second-line | First-line |  |
| 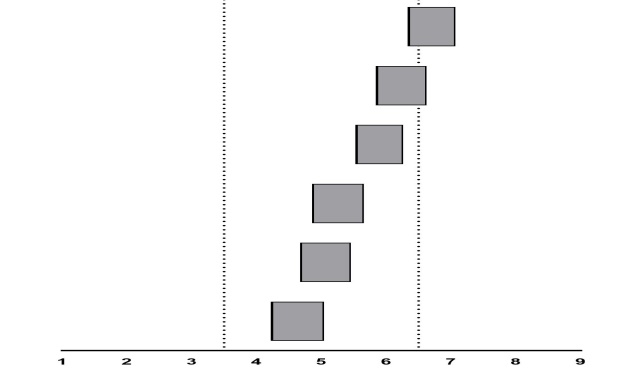Anticipation of physical or mental deterioration |  | | | 6.7 (2.0) |
| History of relapsed anxiety symptoms |  | | | 6.2 (2.1) |
| No stabilization of physical or mental states or QOL |  | | | 5.9 (2.0) |
| Continuation of monotherapy or low dose |  | | | 5.3 (2.2) |
| Patient desire |  | | | 5.1 (2.1) |
| No reported side-effects |  | | | 4.6 (2.3) |

CI, confidence interval; QOL, quality of life; SD, standard deviation

Q7) Which strategy would you recommend for tapering the dosage of or discontinuing a benzodiazepine anxiolytic drug?

| 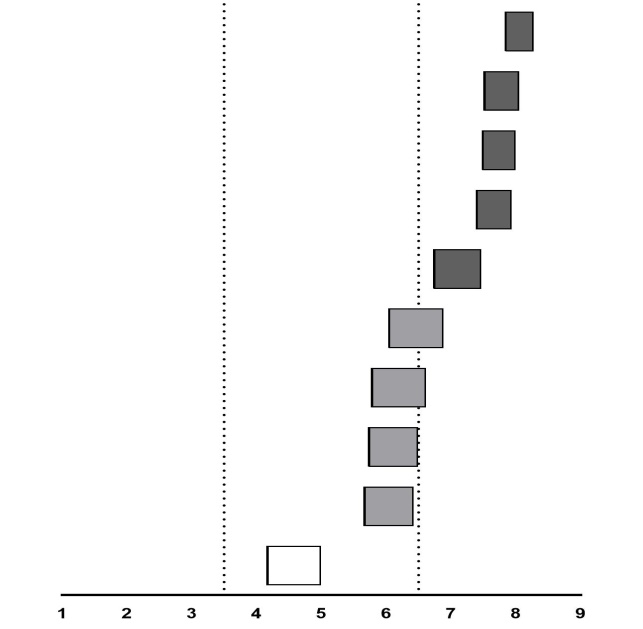 | 95% CI | | | Mean (SD) |
| --- | --- | --- | --- | --- |
|  | Third-line | Second-line | First-line |  |
| Gradual reduction |  | | | 8.1 (1.2) |
| Psychoeducation for anxiety |  | | | 7.8 (1.5) |
| Lifestyle changes |  | | | 7.7 (1.4) |
| Coping strategies |  | | | 7.7 (1.5) |
| Relaxation techniques |  | | | 7.1 (2.0) |
| CBT |  | | | 6.5 (2.3) |
| Mindfulness, attention training |  | | | 6.2 (2.3) |
| Switching to PRN |  | | | 6.1 (2.1) |
| Switching to another drug |  | | | 6.0 (2.1) |
| Self-management |  | | | 4.6 (2.3) |

CBT, cognitive behavioral therapy; CI, confidence interval; PRN, pro re nata (as needed); SD, standard deviation

Q8) Which medication would you recommend switching to when tapering the dosage of or discontinuing a benzodiazepine anxiolytic drug?

|  | 95% CI | | | Mean (SD) |
| --- | --- | --- | --- | --- |
|  | Third-line | Second-line | First-line |  |
| 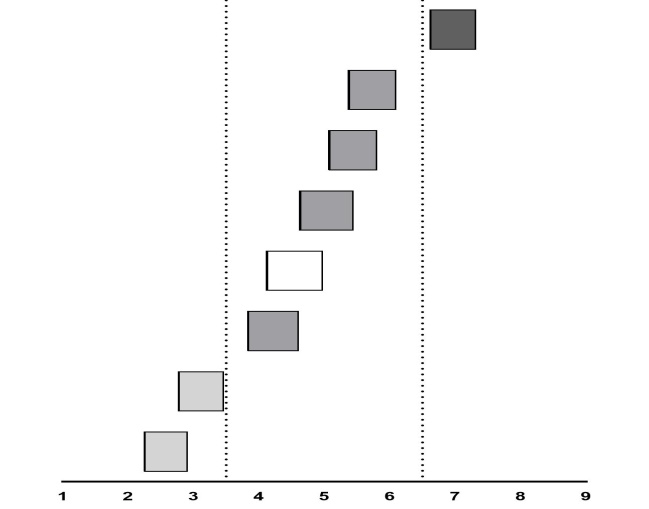SSRIs |  | | | 7.0 (2.0) |
| SNRIs |  | | | 5.7 (2.0) |
| Mirtazapine |  | | | 5.4 (2.1) |
| Tandospirone |  | | | 5.0 (2.3) |
| Kampo |  | | | 4.5 (2.4) |
| Antipsychotic drug |  | | | 4.2 (2.2) |
| Antiepileptic drug |  | | | 3.1 (1.9) |
| Antihistaminic drug |  | | | 2.6 (1.8) |

CI, confidence interval; SD, standard deviation; SNRI, serotonin and norepinephrine reuptake inhibitor; SSRI, selective serotonin reuptake inhibitor
